# Supplementary material for: Activity-induced polar patterns of filaments gliding on a sphere
Source: Nat Commun. 2022 May 11;13:2579. doi: 10.1038/s41467-022-30128-7 (PMC9095588; doi:10.1038/s41467-022-30128-7)
Supplement: Supplementary file 1 — Supplementary Information [file 41467_2022_30128_MOESM1_ESM.pdf]

Supplementary Information for Activity-induced polar patterns of filaments gliding on a sphere

Chiao-Peng Hsu,[1,*] Alfredo Sciortino,[1,*] Yu Alice de la Trobe,[1] and Andreas R. Bausch[1,†]

[1] *Center for Protein Assemblies and Lehrstuhl für Zellbiophysik (E27), Physics Department, Technische Universität München, Garching, Germany*

Purification of gelsolin

Gelsolin was purified from adult bovine serum (Sigma Aldrich). The freshly defrosted serum was supplied with 50 mM Tris-HCl (pH 8.0) and 0.2 mM phenylmethylsulfonyl fluoride. Then, the serum proteins were precipitated with 35% and 50% saturated ammonium sulfate and subsequently spun down by centrifugation. The pellet obtained by the second precipitation was washed twice with a buffer containing 25 mM Tris-HCl (pH 8.0) and 50% saturated ammonium sulfate and then dissolved and subsequently dialyzed against a buffer containing 25 mM Tris-HCl (pH 8.0), 45 mM NaCl, and 1 mM EGTA. Gelsolin was purified by ion-exchange chromatography on a diethylaminoethyl cellulose column by washing first with the same buffer, then with a buffer containing 30 mM NaCl, and finally eluted with a buffer containing 25 mM Tris-HCl (pH 8.0), 30 mM NaCl, and 2 mM $CaCl_2$.

Preparation of biotinylated HMM

Biotin-NHS (Sigma Aldrich) was dissolved in dimethyl sulfoxide (Sigma Aldrich) at 2.5 mg/mL. Biotinylated HMM was obtained by incubating HMM with Biotin-NHS in a 1:10 molar ratio at room temperature for 10 min. The reaction was quenched by adding monosodium glutamate (Sigma Aldrich) in a 4:1 mass ratio (monosodium glutamate to HMM) into the mixture. The biotinylated HMM was then subsequently dialyzed against a buffer containing 5 mM NaH_2PO_4 (pH 6.5), 40 mM NaCl, 2 mM DTT, and 0.8 mM NaN_3.

F-actin length analysis

The gelsolin-mediated fluorescent actin filaments were imaged using total internal reflection microscopy (TIRF; DMi8 S module Infinity TIRF, Leica) with an HC PL APO 100x/1.47 CORR TIRF oil immersion objective. The filaments were imaged in a glass chamber that consisted of cover-slips (Carl Roth) fixed to microscope slides (Carl Roth) by 4-layer parafilm. The cover-slips were coated with a 0.1% nitrocellulose solution, which was made by diluting a 2% solution (Electron Microscopy Sciences) in amyl acetate (Roth), and were left to dry overnight. HMM was diluted in assay buffer (pH 7.4) containing 25 mM Imidazole, 4 mM $MgCl_2$, 25 mM KCl, and 1 mM EGTA. The glass chamber was briefly incubated with the HMM dilution and the surfaces were then passivated with a BSA solution (10 mg/mL dissolved in assay buffer), before the addition of fluorescent-labeled F-actin diluted in assay buffer. The ATP-free conditions allow filaments to bind to HMM in the rigor state. The filaments' length was determined using Ridge Defection in Fiji/ImageJ which exhibiting a log-normal distribution and having the mode length $L = 0.55$ μm (Supplementary Figure 1).

Calculation of F-actin surface density and surface packing fraction

The surface density of F-actin on the inner leaflet of the vesicle was calculated based on the assumption that all the encapsulated F-actin were bound to the membrane. The surface density ρ was obtained by dividing the number of F-actin, $N_{F-actin}$, inside the vesicle by the surface area of the vesicle such that

$$\rho = \frac{N_{F-actin}}{4\pi R^2} = \frac{\frac{4}{3}\pi R^3 \cdot c_A \cdot N_0 \cdot \frac{L_{G-actin}}{L}}{4\pi R^2} = \frac{R \cdot c_A \cdot N_0 \cdot L_{G-actin}}{3 \cdot L}, \quad (1)$$

where R is the vesicle radius, c_A is the encapsulated actin concentration, N_0 is the Avogadro number, $L_{G-actin}$ is the length of each G-actin occupied in an F-actin, and L is the F-actin length. By applying $R = 18$ μm (Supplementary Figure 3), $L_{G-actin} = 2.73$ nm [1], and $L = 0.55$ μm, we obtained $\rho \approx 1.8$, 2.7, 5.3, and 10.7 filaments/μm^2 for $c_A = 100$, 150, 300, and 600 nM, respectively.

The surface packing fraction ϕ was calculated that $\phi = N_{F-actin} Lw/4\pi R^2 = \rho Lw$, where $w = 7$ nm is the width of F-actin. We obtained $\phi = 0.007$, 0.01, 0.02, and 0.04 for $c_A = 100$, 150, 300, and 600 nM, respectively.

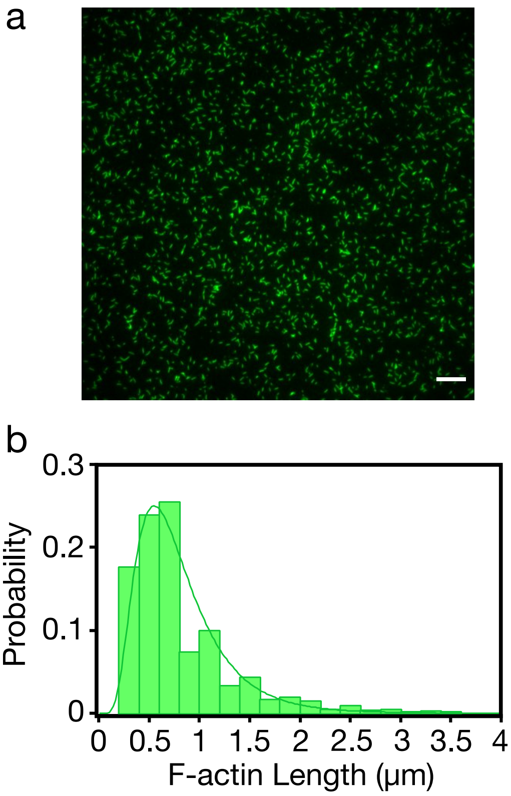

Supplementary Figure 1. **Gelsolin-mediated fluorescent actin filaments.** **a**, TIRF image of the gelsolin-mediated fluorescent actin filaments. Scale bar, 5 μm. **b**, Distribution of the F-actin length, $N = 3745$.

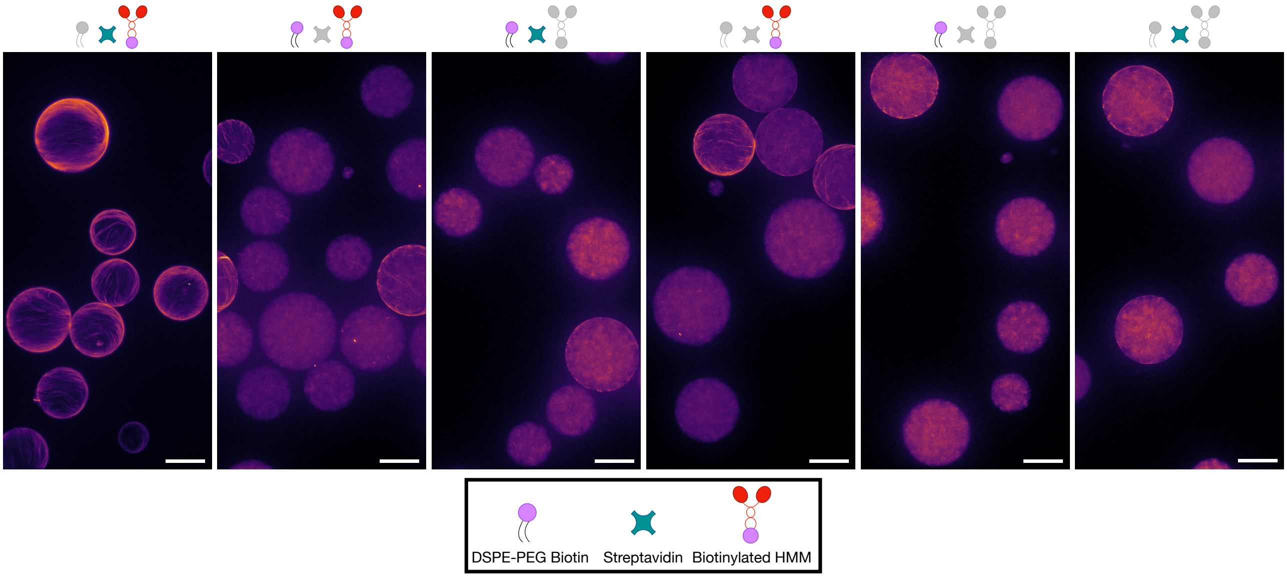

Supplementary Figure 2. **Influence of streptavidin-biotin-interaction components.** Fluorescence images (z-projections) of vesicles with encapsulated actin concentration = 600 nM, but without one or two of the streptavidin-biotin-interaction components after 60 min of production. In all cases, no collective pattern is observed. Scale bars, 20 μm.

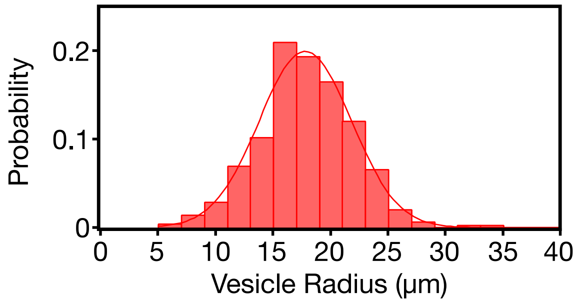

Supplementary Figure 3. **Active actin vesicle size.** Distribution of the radius of active actin vesicles, $N = 492$

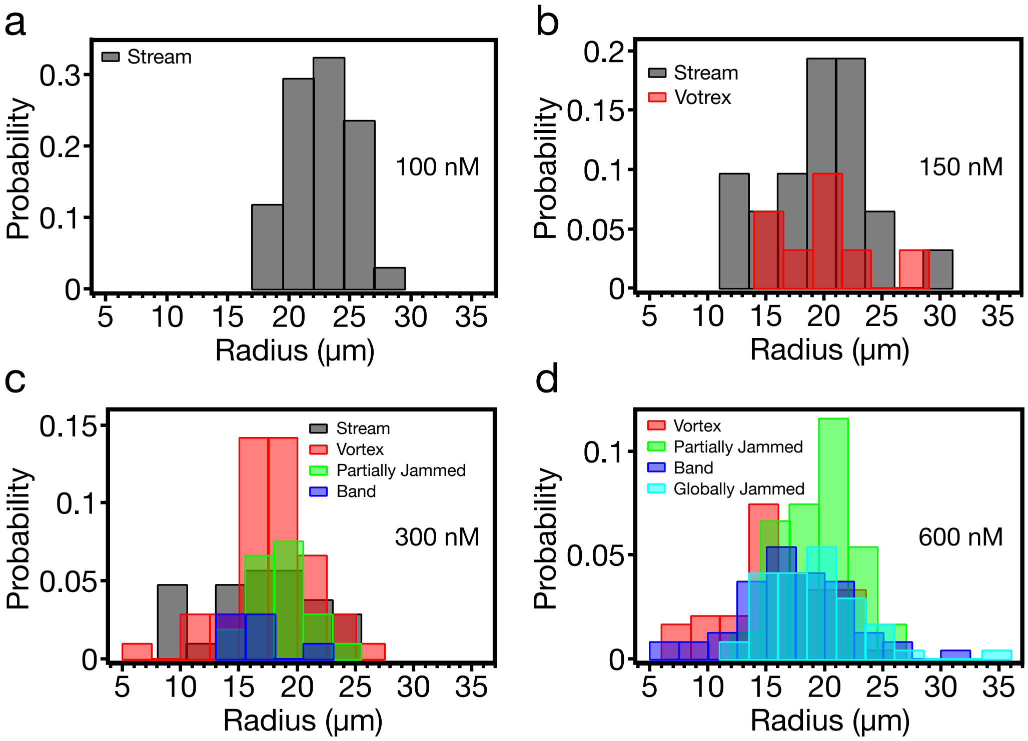

Supplementary Figure 4. **Vesicle size vs. polar patterns. a–d**, The radii of vesicles at 100 nM (**a**), 150 nM (**b**), 300 nM (**c**), and 600 nM (**d**) for each observed polar pattern. The bin width is 2.5 μm.

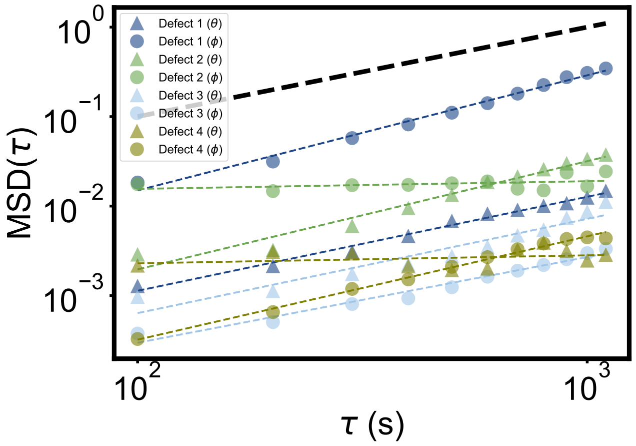

Supplementary Figure 5. **Mean-square displacement (MSD) of globally-jammed patterns.** MSD of the four $+1/2$ defects in a globally-jammed vesicle. Dashed line indicates diffusive behavior (MSD $\propto \tau$).

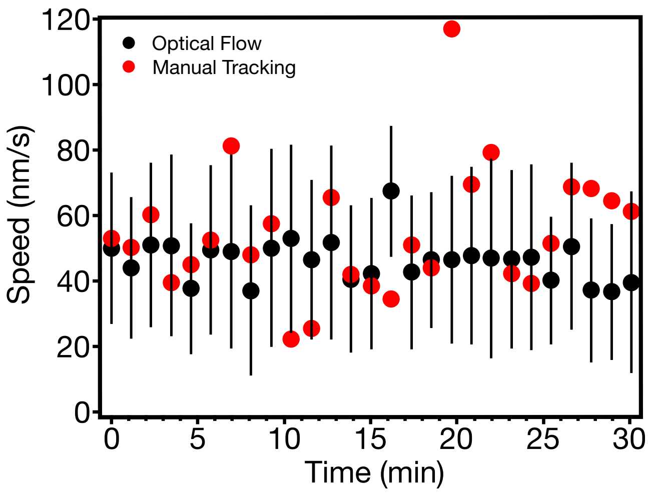

Supplementary Figure 6. **Manuel tracking and optical flow.** The instantaneous speed of a stream obtained by manual tracking (red) is consistent with the one obtained by optical flow method (black). Error bars represent the standard deviations of the velocity extracted from optical flow.

* These authors contributed equally
† abausch@mytum.de

[1] Egelman, E. H., Francis, N. & DeRosier, D. J. F-actin is a helix with a random variable twist. *Nature* **298**, 131–135 (1982).
