# Supplementary material for: Activity-induced polar patterns of filaments gliding on a sphere
Source: Nat Commun. 2022 May 11;13:2579. doi: 10.1038/s41467-022-30128-7 (PMC9095588; doi:10.1038/s41467-022-30128-7)
Supplement: Supplementary file 3 — Description to Supplementary Information [file 41467_2022_30128_MOESM3_ESM.pdf]

Description of Additional Supplementary Information

Supplementary Movie 1:

The formation of steam on a spherical vesicle. The multi-filament collision on the membrane leads to the buildup of a stream at c_A = 100 nM.

Supplementary Movie 2:

The dynamics of single steam on a spherical vesicle. The stream explores the inner surface of the vesicle with a velocity of 40–50 nm/s at c_A = 100 nM.

Supplementary Movie 3:

The merging mechanism on a spherical vesicle. The merging of two streams at c_A = 150 nM where the orientation mismatch leads to the formation of a transient local +1/2 topological defect before the success of merging.

Supplementary Movie 4:

The jamming mechanism on a spherical vesicle. The jamming of two streams at c_A = 300 nM where a local +1/2 topological defect does not resolve after the collision.

Supplementary Movie 5:

The formation of a vortex on a spherical vesicle. Multiple streams collide and merge with other streams and build up a vortex on the vesicle at c_A = 300 nM.

Supplementary Movie 6:

The formation of double vortices on a spherical vesicle. The two long streams are developed at the same time close to each other but travel in opposite directions, which prevents them from merging and builds up two concentric vortices with opposite handedness at c_A = 300 nM.

Supplementary Movie 7:

The formation of a polar band on a spherical vesicle. The length of the stream can be developed to match the circumference of the vesicle leading to the formation of a vesicle-circulating polar band at c_A = 600 nM.

Supplementary Movie 8:

The globally jammed pattern on a spherical vesicle. The activity of actin filaments is largely reduced when arrested in a globally jammed state and the globally jammed pattern evolves very slowly at c_A = 600 nM.
